# A List of the Principal Medicinal Plants Enumerated by Botanists, and Known to Be Growing in Northern Illinois and Wisconsin; with a Slight Notice of Some of Their Medical Properties

**Published:** 1855-02

**Authors:** Stephen W. Williams

**Affiliations:** Late Professor and Lectures upon Materia Medica, Medical Botany, in Dartmouth, N. H., Medical College, and in the Medical University of Willoughby, Ohio, formerly of Deerfield, Massachusetts, now of Laona, Winnebago County, Illinois


### THE NORTH-WESTERN

### MEDICAL AND SURGICAL JOURNAL.

### NEW SERIES.

VOL. IV. FEBRUARY, 1855. NO. 2.

### ORIGINAL COMMUNICATIONS.

ART. I.—*A List of the Principal Medical Plants enumerated by Botanists, and known to be growing in Northern Illinois and Wisconsin*; with a slight notice of some of their medical properties. By STEPHEN W. WILLIAMS, M.D., late Professor and Lectures upon Materia Medica, Medical Botany, in Dartmouth, N. H., Medical College, and in the Medical University of Willoughby, Ohio, formerly of Deerfield, Massachusetts, now of Laona, Winnebago County, Illinois.

The study of Medical Botany is now engaging the attention of medical men throughout the United States. It is very far from being confined to the self-styled botanical physicians, or empirics, as they are emphatically and most appropriately called, who rarely take the trouble to investigate the properties of any plant. They derive most, if not all their boasted information on this subject from the researches of regular physicians.

The resolution adopted by the American Association at Philadelphia, in May, 1847, turned the attention of American physicians to this subject. A committee was appointed, with N. S. Davis, M.D., as chairman, who made a short report upon it, which was followed by two long reports, the one by Francis P. Porcher, M.D., of Charleston, S. C., containing 174 pages royal octavo, and giving an account of more than five hundred medical plants growing in the neighborhood of South Carolina; the other by myself, in a communication containing a list of about three hundred and twenty indigenous medicinal plants growing in Massachusetts, giving an account of their medical properties. Others have given lesser reports in the transactions of the Association, and the medical periodical publications of the day, showing that a growing interest is felt on the subject by our educated physicians.

The field for observation on this subject is vast, and as yet but little explored, particularly in the boundless West, which, comparatively speaking, is just beginning to be populated. The studies of physicians more naturally incline them to the cultivation of the science of natural history, than those of any other class of men; consequently they have done more towards enlarging its boundaries than any other. Their rides in thickets and forests, and in the open grounds, give them great opportunities for investigating this branch of knowledge.

Suffer me in this place to give an extract on this subject from the address of the father of American physicians, the immortal Rush, delivered before his class in the University of Pennsylvania in the year 1789, nearly sixty six years ago. It loses none of its value or significance by age. It also gives a spur and impetus to exertion in other branches of knowledge.

"Let me recommend to your particular attention the indigenous medicines of our country. Cultivate or prepare as many of them as possible, and endeavor to enlarge the materia medica, by exploring the untrodden fields and forests of the United States. The ipecacuanha, the Seneka and Virginia snakeroots, the Carolina pink-root, the spice-wood, the sassafras, the butternut, the thoroughwort, the poke, and the strammonium, are but a small part of the medicinal productions of America. I have no doubt but there are many hundred other plants which now exhale invaluable medicinal virtues in the air. Examine likewise the mineral waters, which are so various in their impregnation, and so common in all parts of our country. Let not the properties of the insects of America escape your investigation. We have already discovered among some of them, a fly equal in its blistering qualities to the famous fly of Spain. Who knows but it may be reserved for

America to furnish the world from her productions, cures for some diseases which now elude the power of medicine? Who knows but at the foot of the Alleghany mountains there blooms a flower that is an infallible cure for the epilepsy? Perhaps on the Monongahela, or the Potomac, there may grow a root that shall supply by its tonic powers the invigorating effects of the savage or military life, in the cure of consumption. Human misery of every kind is evidently on the decline. Happiness, like truth, is an unit. While the world, from the progress of intellectual, moral, and political truth, is framing a more safe and agreeable abode for man, the votaries of medicine should not be idle. All the doors and windows of the temple of nature have been thrown open by the convulsions of the late American revolution. This is the time, therefore, to press upon her altars. We have already drawn from them discourses on morals, philosophy, and government, all of which have human happiness for their object. Let us preserve the unity of truth and happiness, by drawing from the same source, in the present critical moment, a knowledge of antidotes to those diseases which are supposed to be incurable."

For my knowledge of the location of the principal part growing in this section of the country, I am more indebted to a manuscript containing a list of the plants of Wisconsin, by my friend Dr. S. Lathrop, recently Professor in the Beloit College, now a Professor in the Wisconsin University at Madison, than to any other source. I believe this catalogue has recently been published in some of our western periodical journals. I have also received valuable information from Lapham's Hist. of Wisconsin. I also avail myself of my own observations in a more limited sphere, during a short residence in this section of the country. The accounts of the medical properties of the plants enumerated, is from my own researches into the various authorities which have presented themselves to my notice, and this has been the principal labor of the undertaking. After all, but little new may have been elicited, but it will serve as a condensed reference to the medical plants of this section of the west. I have omitted the names of my authorities for the medical properties of the plants enumerated, as it would extend my communication to an indefinite length. I refer the artificial classification of the plant of the Linnian arrangement thus, for the sake of abbreviation, Class 1. Order 13. I say, 1, 13. And for the natural order for Rubiaceæ, for instance, I say, N. O. Rubiaceæ, &c. I trust that most physicians have botanical works describing the plants in their particular locations, and it will be easy from this arrangement to refer to them. I have prefered the alphabetical arrangement to the natural or Linnian one, on account of the facility it gives in looking for the plant.

1. Abies Balsamica. Balsam of Fir. 20, 16. N. O. Conifiræ. This beautiful tree, one of the most beautiful in the American forests, and one which should adorn our door yards more frequently than it does, yields a pellucid balsam, which is expectorant, vulnerary, and slightly stimulant. One part of this balsam, and two of spirits of turpentine forms an elegant transparent varnish for pictures, and paper.

2. Abies Canadensis, Hemlook tree. Class and order as above. Stimulant; branches used in fomentations—used in tanning.

3. Abies nigra. Black Pine, Spina, class and order as above, stimulant, diuretic. The oil of Spina is obtained from this.

4. Abies or Pinus Strobus. White Pine, class and order the same. Most valuable for timber, the bark used for poultices in the piles, stimulant.

The genus Abies taken from Pinus. Except in the pineries of Wisconsin, pines are scarce and timber high.

5. Abutilon Avicennæ. Indian Mallows, N. O. malvaccæ, emollient.

6. Acer Saccharinum. Sugar Maple, Mountain Maple, 8, 1. Its principal medical quality is the sugar which it yields.

7 Acer Rubrum. Red Maple, 8, 1. A good ink is made from it by boiling with sulphate of iron. Decoction useful in sore eyes.

8, 9. Actæa Rubrum et Alba, Cohosh, 13, 1, N. O. Ranunc., tonic, expectorant and nervine.

10. Achillea Millefolium. Yarrow, milfoil, N. O., Artracii. Anti-spasmodic and tonic.

11. Acarus Calamus. Sweet Flag, 6, 1, N. O. orontides. Aromatic, stimulant, used in dyspepsia and as bitter tonic.

12. Adiantum Pedatum. Maiden hair, rock fern, cryptogamia. Aromatic and slightly astringent. A soothing syrup for coughs is made from this plant somewhat similar to the syrup de capillaris.

18. Anemone Nemerosa. Wood, anemone.

19. Anemone Virginica. Wind Flower.

20. A. Pennsylvanica, Anemone. 13, 13, N. O. Ranunc. Properties alike, used in cutaneous affections. It is acrid and irritating, and sometimes used as a substitute for Spanish flies.

21. Amphiocarpæa Monarca. N. O. Liguminous, esculent.

22. Agrimonia Eupatoria. Common Agrimony, 12, 2, tonic, laxative and febrifuge.

23. Alnus Serrulata. Common Alder, 20, 4, N.O. Amentiaciæ. Leaves bitter and astringent. I have found a decoction of the bark of this shrub a most valuable remedy in Hæmaturia. It is also good in other hemorrhages.

24. Alnus Incarnata. Same class and order. Astringent.

25. Allium Canadense. Wild Onion. 6, 1, stimulant diuretic, and laxative. Similar in its properties to the common onion.

26. Amaranthus Altissimus. Prince's Feather, } 20, 5, Both
27. Amaranthus Hybrides. Lovely bleeding, }

these plants are considered to be useful in the hemorrhages, particularly in the uterine hemorrhage.

28. Alisma Plantago. Great Water Plantain, 8, 13, irritant; stimulant. It was formerly considered in Russia to be almost a specific in hydrophobia, but it is not now much used for this purpose.

29. Ambrosia Trifida. Rich weed. This weed was used by the Indians to make ropes. Employed in after-pains in labor, nervine.

30. Arctium Lappa. Burdock, 6, 1. Diaphoretic, diuretic, sudorific.

31. Andromeda Polifolia. Andromeda. Irritant, errhine.

32. Aquilegia Canadensis. Columbine, 13, 5, N. O. Ranunc. Laxative, diuretic.

33. Apios Tuberosa. Ground Nut, 17, 10, N.O. Leguminosa. Esculent, somewhat similar to the potato.

34. Aralia Nudicaulis. Spikenard, 5, 5, true spikenard.

35. Aralia Racemosa. False Sarsaparilla, 5, 5.

Both these species are mild stimulants, aromatic, etc., expectorant. A syrup of these plants is much used in coughs. The bruised root in wounds and old ulcers.

36. Aralia Spinosa. Prickly Ash. Class and order the same. Highly stimulant, irritant, diuretic. Used much in rheumatism, toothache, etc.

37. Arabis Canadensis. Cress. Stimulant, edible.

38. Archangelicæ atropurpurea. Archangel, 5, 2, N.O. Umbellif. Sometimes mistaken for Cicuta or poison hemlock, as it is somewhat similar in appearance. Carminative, stimulant, stomachic.

39. Artemesia canadensis. Wormwood, 18, 2, n. o. corymbifera. The powder of the root has been much used in epilepsy, a most valuable discutient, and much used in fomentations, in wounds and bruises.

40. Arbutus uva ursi. Bear berry. Uva ursi. 1, 10, n, o, Ericæ. Diuretic, tonic, astringent.

41. Apocynum androsæmifolium. Ipecac. Dog's bane. 5, 2, n, o, Apocyncæ, emetic, cathartic, &c.

Apocyncæ cannabinum. Indian hemp, 5, 2. n, o, Apocyncæ, Emetic, cathartic, and diuretic.

42. Asclepias, cornuti. Milk weed, Silk weed, 5, 2, } Emetic.
43. Asclepias incarnati. Silk weed, 5, 2. } Cathartic.

44. Asclepias tuberosa. Pleurisy root, butterfly weed, 5, 2, Pectoral, carminative.

45. Asarum Canadense. Canada snake root, wild ginger, 12, 1. Aromatic stimulant, and diuretic.

46. Arum tryphyllum. Wild turnip, Dragon root, 20, 13. n, o, Aroideæ, expectoront, carminative, diuretic. The root loses much of its causticity in drying.

47. Asplenium thalictroides. Spleen root. cryptogamia. astringent, pectoral, and diuretic.

48. Aster macrophyllus.

49. Aster macrophyllus, } Aster, star flower, 8, 2, n, o, Asteracæ
50 Astor Nor-Angleæ, } Used in cutaneous eruptions, and as an ecbolic.

51. Avena Strictus. Wild oats, 3. 2, n, o, graminacæ, dietetic, expectorant.

52. Asparagus officinalis. Asparagus, 6, 1, n, o, asparagi, dietetic, and diuretic.

53. Betula papyraceæ. White birch, 20, 13, n, o, amentacæ, astringent, diuretic.

54. Baptisia tinctoria. Indigo plant, n, o, Leguminosæ, emetic, cathartic.

55. Brassima peltata. Water shield, 2, 1, mucilaginous, like the lichens.

56. Bidens fondosa, } 18, 2, n, o, corymbiferæ. An infusion of
57. Bidens cernua, } seeds formed into a syrup with honey, good in hooping cough.

58. Bronun ciliatus. Chess, 2, 2. A noxious plant among wheat. Purgative, sudorific, and diuretic.

59. Botrychium fumariordes. Rattlesnake fern, cryptogamia. Mild astringent,

60. Calla Palustris. Water arum, 20, 13. Acrid, stimulant like the wild turnip. It loses its causticity by drying, and is then eaten in Sweden for bread.

61. Caltha palustris. Cowslip, 13, 13, n, o, Ranunculaceæ. Esculent when boiled young in the spring; later acrid. It somesometimes kills cattle.

62 Cardamine rhomboidum. L. Ladies smock. Roots, purgative, diuretic, and nervous, cardamine pratensis.

63. Callytriche verna. Water chickweed, n, o, callitrichiana. Highly diuretic, and useful in dropsy.

64. Canna alba. Perhaps the Juglans, butternut, n, o, Juglandicæ. From the butternut a most valuable cathartic is extracted, which is much in use in domestic practice.

65. Carex arva. Sedge. L. This genus is very extensive. Edible, stomachic, diuretic, especially those with odorous roots.

66. Carpinus virginica, Vel ostrya, n, o, carylaceæ. It affords a grateful food for cattle. The wood is very hard and white, and burns like a candle. It dies yellow.

68. Cassia chamachristus. A species of senna, 10, 1, n, o, Leguminoseæ, cathartic, laxative.

68. Caphalantus occidentalis. Button wood. L. Tonic febrifuge, cathartic.

69. Ceanothus Americanus. New Jersey tea root, 5. 1, n, o, Rhanaceæ. Astringent, used for apthous sore mouth; used as a substitute for tea during the revolutionary war.

70. Celastrus scandens. False. bitter sweet, 5, 1, n, o, celastracean. The bark is emetic and dyscutient. Useful in discussing tumors and swellings in the bags of cows.

71. Celtis occidentalis. Hackberry, 5, 1. N. O. Amentacæ. Anodyne, refrigerant. The berries eaten in dysentery.

72. Cicuta Maculata Wild poison Hemlock, 5, 2. N. O. Umbilliferæ. A narcotic irritant poison like the conium.

73. Cicuta bulbosa. Same class and order. Propertiesthe same.

74. Circæ lutitiana, Enchanted night shade, 1. 2. n. o. Labiatæ, used in enchantments in druidical ages, like the mummery of animal magnetism.

75. Claytonia Virginica. Pigroot. L. Antiscorphulous in cataplasms.

76. Clematis Virginiana. Clematis, 3, 13, n. o, Ranunculacæ. Very acrid, and employed as a caustic. Its fibres make paper.

77. Cimcifuga Racemosa. Cohosh. Black snakeroot, n. o. Ranunc. Tonic, and expectorant. Much used in consumptive complaints.

78. Chimaphyla umbellata. Altered from Pyrola. Spotted winter green. Diuretic and tonic, much used in dropsy.

79. Chilone glabra. Snake head, 14, 2, n. o. Peronata. Bitter, tonic.

80. Chenopodium album. Worm seed. Jerusalem oak, 5, 2, n, o, Chenopodiacæ. Anthelmintic, expectorant, and emmenagogue.

81. Chenopodium hybridium. Class, order, and properties the same.

82. Crataegus coccinea. Thorn bush, 12, 2. Astringent, anti-emetic, stomachic.

83. Conium maculata. Poison hemlock, 5, 2, n, o, Unbelliferæ. Anodyne, acrid, and extremely poisonous. I have seen the best effects from this in cancerous ulcerations, from using the bruised leaves in poultices.

84. Cornus alternifolia,

85. Cornus circinata,

86. Cornus seriaca,

88. Cornus paucifolia,

89. Cornus Canadensis,

} 4, 1, n, o, Hedera. Different species of Dogweed. All tonics, and pretty good substitutes for Peruvian bark. Cornein has been made from Cornus Canadensis, little inferior to Quinine.

90. Coptis trifoliata. Gold thread, Mouth root, 13, 13, n, o, Ranun. Tonic, stomachic, and detergent. Much used in the apthous sore mouths of children.

91. Coreopsis. Tickseed. L. Used as a red dye by the Indians.

92. Corylus Americana. Hazel-nut, 20, 13, n, o, Amentaciaæ. The fruit is said to be good in inflammation of the kidneys. The oil is used in toothache.

93. Clintonia borealis. L. Vulnerary in a decoction of the leaves.

94. Chara vulgaris. Water feathers. L. Anti-spasmodic and vermifuge.

95. Convallaria multifloria. Great Solomon's Seal, 6, 2, n, o, Asparagin. I have seen the best effect in hemorrhoids or piles from a syrup made of four ounces of the dried root of this plant, one quart of water, simmered to a pint, add a pint of molasses and simmer. Dose a wine glass full three times a day.

96. Chrysosplenium. Water carpet. L. Aperient, corroborant.

97. Cuscuta gronovir. Dodder, 5, 2, Bitter, and astringent.

98. Cipressus thyoides. White cedar. L. Infusion of the wood stomachic. The oil used in rheumatism.

99. Cynoglossum officinale. Hounds tongue, 5, 1. Demulcent and sedative. Used in Nœmaphysis.

100, Cypripedium pubescens.

101, Cypripedium parviflorum,

102, Cypripedium spectabile,

} Ladies slippers, 19, 2, n. o. Orchideæ. All most used as a nervine by the botanic physcians and quacks as an anti-spasmodic and nervine. They call it Nervine, America Valerian. I can see but little efficacy in it.

103, Cyprus oleandrus. Bull rush; cryptogamia, diuretic.

104, Datura stramonium. Common Thorn apple, apple penn, 5. 1, n. o. Solaneæ. This is one of the most powerful of our narcotics, and an acrid poison.

105, Diervilla trefida. L. Yellow diervilla; antisyphitic, diuretic.

106. Delphinium consolida. Larkspur, 13, 3, n o. Ramunc. Vulnerary, anti-spasmodic, diuretic.

107, Dentaria laciniata. Tooth root. L ; aromatic and stimulant.

108, Dirca palustris. Leather wood, Moose wood, 8. 1, n. o. T. Thymeleæ. It is emetic, cathartic, rubifacient, and epispastic.

109. Drosera rotundifolia. Linden, L. Pectoral, and used in asthma.

110, Elymus Virginius. The seeds eaten like bread.

111, Equisitem arvense. Horse tail, } Cryptogamia ; both of
112, Equis. hyemale. Scouring rush. } them excellent diuretics

113, Erythronium dens canis. Adder's tongue, 6, 1, n. o. Libiar. The root when dry is farinaceous ; when green, emetic, acrid and stimulant.

114, Euonymus atropurpurea. Spindle tree, L. Fruit emetic, diuretic, in powder good for the itch.

115, Eupatorium perfoliatum. Thoroughwort, boneset, 8, 1, n. o. Compositæ. This is undoubtedly one of our best indigenous cathartics; the flowers are also tonic and emetic.

116, Eupatorium purpureum Joepye root, class and order the same, highly diuretic.

117, Euphorbia maculata. Blooming spurge, 11, 3 ; cathartic, emetic, and stimulant.

118, Fagus ferruginea. Beech, 20, 13. It yields a pleasant edible nut. The decoction of the leaves, and in ointments, good for burns and scalds.

119, Flœrkia proserpin cardis. Sweet salad ; edible, good and sweet.

120, Fragaria virginiana. Strawberry, } 12, 13, n. o. Rosacæ. Pleasant fruit a de-
121, Fragaria vesca, common ditto, } coction of the leaves useful in dysentery.

122, Fedia fago pynum. Lamb lettuce. Mx. A good salad, diuretic.

123, Fraxinus Americana. Ash tree, 21, 2, n o. Jasmineæ. The bark is bitter and astringent, and used for hemorrhages, and the bites of snakes.

124, Galium aperine. } Goose Grass, 4, 1, n. o. Rubiceæ,
125, Galium asprellum, } diuretic, and astringent.

126, Gaultherius procumbens. Winter green, chequer berry, 10, 1. A very pleasant aromatic stimulant. The oil and essence very much used in this country, diuretic.

127, Geranium maculatum. Crane's bill, 16, 10, n. o. Geranaceæ. This is one of our best and purest astringents.

128, Geranium robertianum. Same class and order. Used in the bloody water and bloody flux of animals, astringent and diuretic.

129, Gerandia quircifolia. Golden oak. Mx. Used by the Sioux Indians for the bite of the rattlesnake. It is also used for the toothache.

| | |
|---|---|
| 130, Gentiana quinqueflora.<br>131, Gentiana crinita,<br>132, Gentiana Saponaria. | Blue and fringed gentians, 16, 10, n. o Gentiana, tonic, bitter, corroborant, cathartic, good substitutes for foreign gentians. |

133, Geum robertianum. Avens. Throat root, 12, 3, n. o. Rosaceæ; astringent and a pure tonic; used with gold thread for apthous ulcerations, in chronic diseases, diarrhœa, leucorrhœa, &c.

134, Gnaphalium polyaphelata, Life everlasting, 18, 2, n. o. Corimbiferæ; astringent, vermifuge, and vulnerary.

135, Goodyera pubescens, Scrofula weed. Used much in scrofulous affections.

136. Hydrastis canadenses. Scrofula weed. Much used by the botanists and other empirics. Bitter, pungent, nauseous and disagreeable.

137, Hepatica triloba. Liver root, 13, 13, Ramunc. Expectorant. It has been very extensively used in syrup in consumptive complaints.

138. Helonias deplexa. Helonias. The decoction of the bark of the root useful in colic.

139, Helianthus rigidus. Sunflower, 18, 3, n. o. Corymbifereæ.

140, Helianthus annuus. Large sunflower, class and order the same. The seeds of this plant yield an oil equal to olive oil; astringent.

141, Helianthus tuberosus. Artichoke, class and order the same. Culinary roots, which contains sugar, diuretic.

142, Helianthus canadenses, properties somewhat similar.

143, Helenium autumnale. False Sunflower, yellow star. R. Tonic, febrifuge, errhine. It has been used in intermittents.

145. Heracleum lanatum. Martin root, 5, 2, n. o. apiaceæ; a warm stimulating carminative, like the anise and caraway.

145, Hieracium canadenses. Vining hawkweed, 18, 1, n. o. cichor. vulnerary, astringent, sudorific, and pectoral.

145, Hieracium gronovir, class and order the same; used for tooth ache, and to cure warts.

147, Hordeum jubatum. A species of barley; used like the genuine.

148, Humulus lupulus. Common hop, 21, 5. Bitter, tonic, narcotic, lupulin, or the powder of the hop easily separated from it. They are the principal ingredients in beer and porter.

149, Hydrophyllum virginicum. Burn flower, 5, 2. This has been used against the bites of snakes, and that erysipelatous inflammation of the skin produced by the Rhus or poison sumach.

150, Hypericum pyramidatum, } St. John's wort.
151, Hypericum canadense, } St. John's wort, 5, 1; Balsamic, pectoral, somewhat styptic and vulnerary. Used in diarrhæa, dysentery, mania and low spirits; a syrup of it useful in croup.

152, Impatiens pallida. Jewel weed, 5, 1. The whole plant is acrid, and when taken internally it operates as an emetic, cathartic and diuretic.

153, Iris versicolor. Flower de luce, } 3, 1, cathartic, acrid,
154, Iris viginianum. Blue flag lily. } stimulant and emetic.

155, Juglans cinerea, Butter nut, } class 20, order
156, Juglans cinerea, Black walnut, } 13. The butter-
157, Juglans squarrosa shag bark hickory nut } nut is a most valuable cathartic. The bark of the others is rubifacient.

158, Juncus valticus. Rushes, L. diuretic, cathartic

159, Juniperus communis. Common Juniper, } 21, 16, stimu-
160, Juniperus virginianum. Red cedar, } lant, emenagogue, diuretic, and diaphoretic.

161, Kalmia glauca. Laurel, 10, 1, leaves poisonous and narcotic.

152, Larix Americana. Larix. Hackmetac, 20, 16. It produces a fine balsam similar to turpentine, useful in wounds, bruises, &c. This tree resembles the pinus, but the leaves are deciduous in the fall.

163, Ledum latifolium. Labrador tea, Marsh tea. Leaves bitter, astringent, and pectoral; used in cutaneous eruptions.

164, Lemna minor. Duck meat, 20, 2. A cataplasm of it has been used in gout, and in the piles. A decoction useful in Jaundice.

165, Leonorus cardiaca. Motherwort, 14, 1. Similar in its properties to valerian; used in hysterical and nervous effections.

166, Leontodon taraxacum. Dandelion, 18, 1. This plant has come into vogue greatly for jaundice, dyspepsia, &c. It is diuretic, slightly tonic, and aperient.

167, Lepidium virginianum. Pepper grass, 15, 2. Eaten as a salad; acrid, diuretic, anti-scorbutic.

168, Lilium canadenses. } Canada and Philadelphia lily, L.
169, Lilium philadelphium; } Roots useful in suppuration for poultices. Edible.

170, Liatris cylindrica. Gay feather, 1[illegible], 1. Diuretic; good in gravel.

171, Liatris scariosa. Button snake root, class, order and properties the same.

172, Linum rigidum. Wild flax.

173, Linum usitatissimum, common flax naturalized, 5, 5, laxative pectoral, sudorific, flax seed, one of our purest demulcents.

174, Lobelia cardinalis, Cardinal lobelia, 5, 1.

175, Lobelia syphilitica. Syphilitic lobelia, 5, 1.

176, Lobelia inflata. Devil's pepper, 5, 1. The last article is the divine remedy of the Thomsonian, Botanics, Steamers, and Eclectics, which may all be ranked under the same class, and the one on which they raised themselves to their little ephemeral popularity, and one which with their short lived popularity descended almost to the shades of oblivion. It is a violent narcotic, acrid poison, and highly irritant I have given a full account of it in the New York Journal of Medicine and Surgery, for the year 1846.

177, Lonicera sempervirens. Honeysuckle, 5, 1. The ripe berries of this beautiful plant are strongly purgative. The leaves and flowers are bitterish, mucilaginous and detersive. A syrup is prepared from them for sore throat and irritability of the lungs.

178, Lupinus perennis. Lupini, Finger leaf, L. Seeds bitter and flatulent.

179, Lycopodium lucidulum. Ground pine, L. Cryptogamia; used in dropsy, emenagogue, drastic, gout, diarrhœa, &c.

180, Lycopus virginicus Water agrimony. Bugle 2, 1. This is one of our most valuable astringents: very valuable in uterine and all other hemorrhages. I have published a long account of it in the New York Journal of Medicine. I hope it will continue to engage the observation and attention of physicians.

181, Lycopus minatus. Water horehound, class and order the same. Used in intermittent fevers.

182, Lythrum alatum. Willow herb. L. Mucilaginous in diarrhœa and dysentery.

183, Lysimachia quadrifolia. Loose strife. L. Slightly astringent, stomachic, expectorant; good for coughs and cold, and to improve the appetite.

184, Lythospernum officinale. L. Similar in properties to cynoglossum.

185, Malva rotundifolia. Mallows, class 17; all the mallows are mucilaginous, and equivalent in properties to the slippery elm, gum arabic, &c.

186, Marula cotula. May weed. L. Bitter, tonic, stomachic, nervine.

187, Marubium vulgarie. Hore hound, 14, 1; emenagogue, deobstruent, diuretic, used in dysmenorrhœa, and in affections of the kidneys. It is the basis of the negro remedy for the bite of snakes.

188, Medeola virginica. Indian cucumber, 6, 3. It is highly diuretic, and has been successfully used in dropsies.

189, Menispermum canadense. Moonseed, L. Bitter, tonic, mucilaginous; used in the stranguary of horses.

190, Mentha canadenses. Common mint, 13, 1, n. o. Labiatæ; all the mints are warm aromatic stimulants. The wild leaves applied warm on the breast will prevent the formation of abscesses, and promote the flow of milk.

191, Meriganthus trifoliata. Buck bean, 5, 1, n. o. Gentianæ, Bitter, tonic, and cathartic. More intensely bitter than gentian; used in intermittent and other fevers, where tonics are required; in rheumatism, dropsy, and cutaneous eruptions.

192, Mitella dyphylla. Currant leaf; Refrigerant, used in fevers as a drink.

193, Mitchella repens. Creeping chequer berry. This is a most valuable diuretic, much used in dropsy. For an extended account of it see my Medical Botany.

194, Monarda fistulosa. Balm, 2, 1, n. o. Labiateæ.

195, Monarda punctata. Mountain Balm, 2, 1, n. o. the same; altered to Milissa. Aromatic, fragrant, and anti-emetic, similar to the mints.

196, Monotropa Uniflora. Beech drop, pipe plant, 10, 1, N. Monotropæ. Anodyne. Dr. Steward's substitute for opium; cures opthalmies.

197, Milium effusum. Wild Millet. L Esculent.

198, Myrica gala. Bay berry. n. o. Myricæ; astringent, anti-spasmodic used in dysentery. From the berries bay-berry tallow is made.

199, Nasturtium palustre, Nasturtion, L; aromatic, diuretic.

200, Negundo acerandis. Equivalent with all the maples.

201, Nymphæa odorata. White pond lily, sweet pond lily, 13, 1. The roots are the parts employed in medicine. They are demulcent, anodyne and emollient. They form the best poultices which can be used in suppurating abscesses.

202, Nuphar advena. Yellow water lily, 13, 1. This plant possesses the properties of the above, but in a less degree.

203, Nepeta cataria. Catmint or catnep, 5, 1, n. o. Labiatæ. carminative, stimulant, and much used in the affections of infants.

204, Oxalis violacæ. Clover leafed sorrel, L. 1, 5. This plant yields oxalic acid.

205, Œnothera biennis. Scabish Tree primrose, 8, 1, n. o. Malastron; esculent. mucilaginous.

206, Orchis spectabilis, Salep, L. From this Ocher's Salip is made, vermifuge.

207, Ostrya virginica. Similar to carpinus.

208, Oryzoptis asperifolia. American rice; eaten by the Amrican Indians like common rice.

290, Panax quinquefolia. Ginseng, 5, 2. The divine remedy of the Chinese. Very abundant here, sufficient for commercial purposes. Stimulant cordial tonic, expectorant, &c. An excellent ingredient with mallows in cough mixtures.

210, Parnassia. Similar in its properties to hepatica.

211, Pastinaæ Saliva. Parsnip, 5, 2. Esculent. Seeds aromatic and stimulant.

212, Pedicularis canadenses. House wort, 14, 2. Sometimes called heal-all. The Indians use it in the bite of snakes.

213, Pinus banksiana. See Abies, 29, 16.

214, Pinus resinosa. Pitch pine, 29, 16. From this the turpentine and resin of commerce is made.

215, Pinus mitis. Pine 29, 16. All the kinds of pines are valuable, from which the pitch, resin, and turpentine are made. Diuretic and stimulant.

216. Plantago major. Common plantain, etc. Leaves cooling and vulnerary. Root astringent. When young, leaves a good salad.

217. Phytolacca decandra. Poke root, 11, 10. Emetic, cathartic, narcotic. Used much in the cure of rheumatism; also in the swelled udder of cows.

218. Physalis viscera. Ground cherry, 5, 1. Berries diuretic and sedative.

219. Podophyllum peltatum. May apple. Mandrake, n. o. Actiana. This is one of our very best native cathartics at the west, and it grows in great abundance there. On account of its superior efficacy as a cathartic, it is sometimes called mercury root. An account of it may be found in all our best medical dispensations. Leaves narcotic. Fruit relished by most people.

220. Partulucca olevacea. Purslain 11, 12. Diuretic and vermifuge. Esculant. A good salve made with it for the lips and nipples.

221. Polygala paucifolia. Dwarf milk-root, 17, 3, n. o. Polygonacæ. Properties somewhat similar to polygala senega, stimulant, sudorific. Smells somewhat like the Gaultheria procumbens. A tea of the plant useful in erysipelas. Used for rattlesnake bites.

222. P. Sanguinia. } Snakeroots. Class and order as above.
223. P. Polygamia. } Properties somewhat similar.

214, Polygonum persicaria. Water pepper, Smart weed, 9, 3, n. o. Polygoniacæ A pungent, biting, aromatic plant, diuretic and stimulating vesicant, and blistering the skin if applied long to it. Used a good deal in fomentation when an irritant effect is desirable.

216, Polygonum hyderpipe, Similar in properties to the above.

216, Polygonum fagopyrum. Buck wheat. Esculent, flour in pancakes equal to wheat. A tea of the plant useful in erysipelas, both as a beverage and wash.

217, Polygonatum pubescens.

218, Populus tremuloides Common poplar. Class 21. This is the celebrated tonic bitter of the Thompsonian or steamers. The buds are balsamic and stimulant.

219. Populus balsamifera. Balsam poplar. The balsam from the buds of this tree is considered to be equal to copaiva.

120, Polygonus grandidenta.

121, Potentilla norwegica. Cinquefoil, fine finger, 12, 13. This is a mild astringent, and was formerly used in diarrhœa. Properties somewhat similar to tormentilla.

122, Prunella vulgaris. Self heal. 14, 1. Bitter astringent. Bruised and applied to wounds supposed to be very effectual in healing them.

123. Prinos verticellatus. Red berry alder. Bark astringent, emetic, and tonic. Berries purgative and vermifuge. A very ornamental shrub, which would add much to the beauty of our door yards.

124, Prunus Amcricana. Wild cherry, black cherry, 12, 1. n. o. Rosaceæ. This is a bitter astringent, and contains Prussic acid. Tonic, antibilious, and pectoral. It is the basis of Ayer's celebrated cherry pectoral.

125, Pyrus coronaria. Wild crab, 12, 1. Blossoms and fruit acrid, bitter and austere, still the fruit is much used in preserves.

126, Pyrola rotundifolia. Round leafed winter green, 10, 1. Diuretic, leaves vulnerary.

127, Pycnanthemum lanceolatum. Wild basil, 14, 1. This plant is similar in its properties to the mints and pennyroyal.

128, Ptelea trifoliata. Wing seed, vermifuge in tea, vulnerary in poultices.

129, Quercus alba. White oak, 130, Quercus rubra. Red oak, 131, Quercus pinus, } 20, 13. All the oaks are valuable astringents. From them tanin is prepared, the most valuable astringent in use.

132, Ranunculus repens. Crowfoot, butter cup, 14, 1. Acrid and narcotic. The leaves applied to the skin will blister it.

133, Ribes cynosbati. Gooseberry, 5. 1. Fruit edible, laxative, refrigerant. Their properties are universally known.

134, Rhamnus alnifolius. A species of buckthorn. Properties somewhat similar to Rhamnus catharticus, class 5, 1.

135, Rhus typhina. Sumach, 136, Rhus glabra. Smooth sumach } 5, 3. Harmless astringent. Berries used in dysentery.

137. Rhus vernata. Poison sumach. 138, Rhus toxi codendendron, Poison sumach } Poisonous, class and order the same. Produces an effect like erysipelas.

139. Rubus occidentalis. Bramble raspberry. 140, Rubus villosus. Blackberry. 141, Rubus nathamis. Perhaps dewberry. } 13, 13. Properties of the above berries and shrubs are too well known for description here.

142, Rumex brittanica, 6, 3, Acid and rifrigerant.

143. Rumex acetosella. Sorrel dock.

134, Rumex Crispus. dock, class and order the same. Astringent, anti-scorbutic, and tonic. Much was formerly thought of its efficacy in cancer.

145, Rosa lucinda. Rose 13, 13, of the more than 300 species of roses, 30 at least are wild in America. Their properties are similar and well known to educated American physicians.

146, Sanguinaria canadenses. Blood root, 13, 1. A volume might be written in praise of this valuable plant. An account of its virtues may be found in all the writers upon Materia Medica and Medical Botany extant. That of Tully, in the Philadelphia Medical Recorder, is the most elaborate, to which I refer the reader.

147. Sambucus canadenses, Common elder, 3, 3. Flowers laxative, and somewhat sedative. The syrup also possesses much the same properties. Much used in the diseases of infants. Good in cutaneous eruptions. The berries make good wine.

148, Sanicula marylandica. Sanicle, 5, 2. sub-astringent, tonic, antisyphilitic. It has been used as a diuretic in cases of dropsy, also in gonorhœa, syphilis. In decoction it is good in wounds and bruises.

149, Sarracenia purpurea. Side saddle flower, 13, 1, n. o. Sarracenaceæ. The roots of this singular plant are bitter, tonic, and stomachic, and are useful in dyspepsia, and where tonics are required. Useful in diarrhœa and chronic dysentery.

150, Saponaria vaccatia. Soapwort, 10, 2. Tonic, diaphoretic, and hepatic. It has been used in rheumatism, gout and jaundice. They are diuretic and emmenagogue.

151, Saxifraga aiton, Saxifraga,

152, Saxifraga Pennsylvanica. Rock saxifraga, 10, 1. They are bitter and astringent. Root useful in gravel.

153, Scipus pungens. Bull rush. Cryptogamia. Diuretic.

154, Scutellara lateriflora. Scull cap, mad dog weed, 14, 2, n. o. Labiateæ. Volumes have been written and more angry passion has been excited upon the anti-hydrophobic virtues of this plant, and much ridicule has been cast upon the believers in it. Still the facts and arguments which have been adduced in its favor have not been overthrown. Some of the most respectable physicians in England, and in the United States have been believers in it, such as Dr. Mussey, of Cincinnati, Dr. Spalding, Drs. Wells, Stone, Williams, Rafanisque, and many others in America, besides Youatt and Watson, in Europe. I am no believer in any specific, yet I think the subject should be treated with candor, rather than ridicule. Surely a substance containing the following ingredients cannot be considered inert. The substances found in it by Cadet were, 1st. Yellow green oil, fixed, and soluble in ether. 2nd. A bitter principle. 3rd. Chlorophylle. 4th. A peculiar volatile matter, tasting and smelling like the principle of anti-scorbutic plants 5th. An essential oil. 6th. Albumen. 7th. A sweet mucous substance. 8th. A peculiar astringent principle. 9th, Lignin. When burnt the ashes afford chloride of soda and seven other salts. It is, says he, tonic, astringent, anti-spasmodic, anti-hydrophobic at least. The steamers and botanics consider it their grand anti-spasmodic. (See my communication in the transactions of the American Medical Assoeiaton, vol, 2, 1849.)

155, Santillaria galimculata. Hooded willow herb, class and order the same. Anti spasmodic.

156, Silphum lanchium. Turpentine gum flower, L. Yields a fine fragrant gum like Frankinsence, and chewed by the Indians to sweeten the breath.

157, Silene stellata. Wild pink, L. vermifuge.

158, Sinapis alba. White mustard, 15, 2. Antiscorbutic and diuretic; leaves used as a salad.

159, Sinapis nigra, Black mustard, 15, 2; heating and stimulant. Used as a condiment. Seeds in large quantities unbroken emetic; bruised most excellent for an external irritant.

160, Sisymbrium canescens. Hedge mustard, n. o. cruciferæ, diuretic, and expectorant.

161, Smilax rotundifolia. Green briar, 21, 6. An infusion is said to be of service in mercurial salivation, in chronic rheumatism, and in diseases of the skin.

162. Spergula arvensis. Spurge, 10, 5. The inhabitants of Norway and Finland use the seeds for bread, when the corn fails; poultry are fond of them.

163, Solidago laterifolia. Golden rod, 18, 2.

164, Solidago odora. Sweet scented golden rod, 18, 2. Carminative. Its properties are very similar to aniseed, caraway, &c., aromatic and stimulant.

165, Sorghum natans, L. Esculent.

166, Spiroœa opulifolia. A species of hard hack, 12, 5, slightly astringent and tonic.

167. Symphonia carpus occidentalis. Snow berry; root used for ague, tonic, and astringent; fibrifuge in small doses.

168, Symplocarpus foetidus. Skunk cabbage, n, o. Irvidian Root acrid and stimulant; when green applied to the tongue it will instantly blister it; very extensively used in medicine, an account of which may be found in all our dispensatories.

169, Thalictrum anemanoides. Meadow rue, 13, 13; a poultice made of the leaves of this plant has been known to alleviate the pain of sciatica.

170, Thalictrum diocum. Same class and order; sometimes used for snake bites.

171, Tiphrosia virginica. Turkey pea, L. Vermifuge.

172, Teucrium canadense. Wood sage, germander, 14, 1. Tonic, aromatic, bitter.

172, Tilia americana, Bass or Linn, 13, 1. This is a most valuable wood. The bark formed into poultices is superior to slippery elm, and used for the same purposes. Most extensively used in burns, bruises and ulcers.

173, Trillium cernium. Nodding trillium, 6, 3. The roots of all the trilliums are most valuable astringents. I have published a long article upon them in the New England Journal of Medicine and Surgery, and in the New York Journal of Medicine, and in the Transactions of the American Medical Association.

174, Triosteum perfoliatum. Fever root, 5, 1. Cathartic, and in large doses emetic, diuretic, useful in fever, agues, and pleurises.

175, Ulmus Americana. Common elm, 5, 2. A splendid ornamental tree. Diuretic and demulcent.

176, Ulmus fulva. Slippery elm, 5, 2. The bark is one of the purest demulcents known for drinks and poultices.

177, Urtica pumila. Common nettle, 20, 4, stimulant, diuretic.

178, Valeriana ciliala. American valerian, L. Used in nervous diseases.

179, Vaccinium macrocarpus The whortleberry species, L, 10, 1, diuretic.

189, Verbascum thapsus. Mullein, L. Much used as an external mild irritant, by binding the leaves on the part effected, demulcent, and pectoral.

181, Verbena hastata. Purple vervain. 14, 1, bitter, emetic, expectorant. A syrup prepared from it very useful in coughs.

182, Viburnum opulus. Snow ball. } 5, 3, diuretic, berries
183, Viburnum lentago. Haw berry, } eaten by partridges and other birds.

184, Viola blanda. Smooth violet, } All the violets are highly
185, Viola cucullata. Hat violet, } mucilaginous and diuretic
186, Viola pedata. Bird foot violet. } I have published a full account of them in the New York Journal of Medicine and Surgery, which has been republished in the American Journal of Pharmacy, Philadelphia.

187, Viola pubescens. Yellow wood's violet.

188, Vitis æstivalis. Summer grape. } 5, I. For an account of
189, Vitis riparia. Frost grape. } the fortynine species of American grape see Rafinisques Medical Botany, vol. 2, p. 121.

190, Xanthium strumarium. Clott burr, 20, 5. Leaves bitter and astringent, useful in scrofula and erysipelas.

191, Zanthoxylon Americana. Prickly ash, 20, 5. An acrid stimulant similar in its properties to the stratias which see. Used in chronic rheumatism, tooth ache, &c.

192, Zizania aquatica. Wild rice, water oats, L. Esculent. Much relished by cattle and horses.

